# AmReS: an observational retrospective time-to-event analysis of staff voluntary turnover in an English ambulance trust

**DOI:** 10.1136/bmjopen-2024-098174

**Published:** 2025-04-15

**Authors:** Robert M Cook, Zillur Rahman Shabuz, Matthew Bennett, Josh Miller, Abigail East, Alisen Dube, Gina Varnals, Md Asaduzzaman, Mark Radford, Alison Leary, Sarahjane Jones

**Affiliations:** 1 School of Health and Social Care, University of Staffordshire, Stafford Campus, Stafford, UK; 2 Lancaster University, Lancaster, UK; 3 London Ambulance Service NHS Trust, London, UK; 4 West Midlands Ambulance Service NHS Foundation Trust, Brierley Hill, UK; 5 North West London Cancer Network, London, UK; 6 Department of Engineering, University of Staffordshire, Stoke on Trent, UK; 7 NHS England, Redditch, UK; 8 London South Bank University, London, UK

**Keywords:** ACCIDENT & EMERGENCY MEDICINE, Burnout, Health Services, Health Workforce

## Abstract

**Abstract:**

**Objectives:**

The purpose of this study was to identify which, and to what extent, demographic and operational factors are indicative of likelihood for a new call handler or paramedic to remain in role within the first two years of employment at an ambulance trust using data held in the trust’s bespoke data warehouse.

**Design:**

The study uses a retrospective observational cohort design using routinely collected data.

**Setting:**

One ambulance trust focused on a large, predominantly urban area in the UK.

**Participants:**

The study used the data of all employees of the trust who started employment as call handlers (869) or paramedics (1672) between 1 January 2018 and 31 July 2023.

**Primary and secondary outcome measures:**

‘Time-to-event’ analysis of ‘likelihood to remain in post within the first two years of employment’ as call handlers or paramedics via accelerated failure time regression.

**Results:**

Several factors showed a significant contribution to the likelihood of remaining in post within an ambulance National Health Service Trust. Among the findings, short-term sick leave in the first two years of employment was associated with increased retention for paramedics (0.040, 95% CI 0.030, 0.060). In addition, female call handlers were found to have increased retention (0.29, 95% CI 0.043, 0.54), and paramedic retention increased with time outside of ‘job cycle time’ (JCT) activities (ie, activities other than responding to calls) (0.097, 95% CI 0.057, 0.14).

**Conclusions:**

This study presents a method for extracting new insights from routinely collected operational data, identifying common drivers and specific predictors for retention among the ambulance NHS workforce. It emphasises the importance of workforce-centred retention strategies, highlighting the need for non-JCT time, which in turn would allow paramedics to have time to reflect and recuperate to avoid burnout and attrition. The study also suggests that a lack of sick leave might indicate a lack of trust and self-care culture, potentially leading to paramedic staff attrition. Our approach to retention analytics provides a new mechanism for trusts to monitor and respond to their attrition risks in a timely, proactive fashion.

**STRENGTHS AND LIMITATIONS OF THIS STUDY**

- This was a single-centre study, with an ambulance trust focused on a high population density urban area. However, the methodology will be transferrable to diverse settings.
- The study is an observational retrospective analysis; hence, findings and patterns found in the data may be correlational, not causational. Operational interventions taken from such findings need to be tracked to confirm the scale of the effect.
- The study has made use of real in situ data reflective of the data and tasks as done. Hence, the replication of the work at other trusts either as one-off insights or as part of their operational oversight is relatively resource inexpensive.
- The study makes use of routinely collected data so translation to different ambulance trusts is straightforward.
- The data continues to be collected, so the analytics can be deployed as a live intelligence tool.

## Introduction

 The National Health Service (NHS) stands as the largest employer in England, employing a workforce of over 1.3 million individuals.^1 2^ As of June 2024, there were over 100 000 job vacancies in the NHS,^1^ and staff shortages have been demonstrated to directly impact the quality and safety of care, patient experience and staff work experience.^3^ The increasing demand following the COVID-19 pandemic poses additional threats to staff retention, patient outcomes and staff well-being,^1 4^ and so workforce retention is a timely priority for the NHS as outlined in the People Plan^5^ and the NHS Long-Term Workforce Plan.^2^

Emergency medical services (EMS) formed the frontline of the COVID-19 response and, particularly in England, are now faced with significant workforce shortages that affect their efficiency and effectiveness. Between 2021/2022 and 2022/2023, the average workforce vacancy rates for the ambulance sector increased from 3.6% to 6.6%,^6^ further exacerbated by reports that at least one in four paramedics have considered leaving their roles due to frustrations with inadequate patient services.^7^ Those remaining in post face growing pressures to deliver a critical service where poor retention has already been linked to high levels of burnout, depersonalisation, heavy workloads and feelings of being unsupported or regularly endangered.^8^,^10^

Demands to complement the current professional healthcare workforce are not novel. Health Education England estimated that the NHS would require to recruit at least twice as many new paramedic trainees each year to meet future demand.^11^ However, simply recruiting more staff risks leaving the underlying issues unresolved, with the subsequent retention of staff potentially affected. Considered within the framework of Herzberg’s motivator-hygiene theory of satisfaction,^12^ the environment into which a new hire arrives is key to the concept of workplace hygiene as mediated via coworker relationships and work environment. Poor ‘hygiene’ leads to growing dissatisfaction within the workplace, and hence, any attempts to create satisfaction via ‘motivator’ mechanisms may go unrealised.

Herzberg’s theory has been central to multiple studies of retention within healthcare, though few studies have focused on the EMS setting.^13^ In the integrated urgent care Workforce Blueprint,^14^ NHS England reflected on the findings of recent staff surveys, noting that while call handlers reported that they ‘feel like they make a difference to patients and service users’ the common hygiene issues of work environment and support were present. Managers and policymakers are aware that overwork increases the prevalence of turnover, but what they do not have is reliable information as to when a staff member is overworking. There is no proactive mechanism to monitor the workforce for individuals at a heightened risk of attrition so they can address risks as they evolve, and hence, we look to address this gap by studying attrition via an ambulance service’s existing operational data.

Routinely collected data can be a valuable resource that complements current commonly used research methods that focus on staff feedback; healthcare providers can leverage readily available big data and specific analytical techniques to understand, monitor and address issues related to workforce retention. Use of such data can provide a comprehensive view and insight into the contributory factors associated with staff turnover over time, and thus, facilitate evidence-based development of retention strategies based on real-time monitoring. Consequently, fostering a positive work environment that ensures a continuity of high-quality care. This study analyses operational data from a major English ambulance service to identify which, and to what extent, demographic and operational factors are indicative of likelihood for a new call handler or paramedic to remain in role within the first two years of employment at an ambulance trust.

## Methods

This is a single-centre retrospective observational study using anonymised routinely collected data from an ambulance NHS Trust in England. The study period was set from 1 January 2018 to 31 July 2023.

### Data preparation

Seven data sets (see table 1) were extracted from the ambulance trust’s data warehouse using bespoke structured query language scripts developed by the research team in collaboration with the ambulance trust’s nominated business analysts. The business analysts were responsible for extracting the data and ensuring it was deidentified before sharing with the research team for analysis. ‘Deidentification’ was performed using an anonymisation technique, replacing free text by a randomly generated alpha-numeric string, which was then reused when the same free text reappeared and was outlined in the ethics application for the study.

**Table 1:** Summary of extracted data sets and variables

Data set	Description	File size*	Variables of interest
Employment records	Periods of employment for each member of staff. Each row represents one continuous period of employment.	2.1 MBN=11 803	Employee ID Start/end dates of ‘employment’ Staff demographics
Historical assignments	Time series of positions for each employee of the trust. Each row represents one ‘assignment’ (a period working in a given post and location) with periods of employment made up of multiple ‘assignments’.	12.1 MBN=1 04 852	Employee ID Start/end dates of each ‘assignment’ Job title Pay band Indication if this was a period of active work or not (non-active assignments including maternity leave and secondments)
Shifts	Time series of rostered and planned overtime shifts of individuals. Each row represents one shift of one individual.	191 MBN=2 714 042	Employee ID Shift start/end time Assigned ambulance callsign (if applicable)
Incidents	Record of emergency service calls responded to by the ambulance service. Each row represents a call attended.	1.3 GBN=11 382 236	Responding ambulance callsign(s) ‘Job cycle times’† Conveyance‡ status Index of Multiple Deprivation decile of response location
Contacts	Record of all calls made to the ambulance service. Each row represents one call to the ambulance service.	548 MBN=11 401 902	Incident ID Incident category
Overtime	Time series of work done beyond the rostered shifts. Each row is one period of overtime for one member of staff	186.5 MBN=2 379 485	Employee ID Type of overtime (planned, unplanned, payments in lieu of breaks, etc) Time spent on overtime
Staff absence/ sickness	Time series of short-term employee absences due to illness. Each row represents one period of sickness for one employee.	4 MBN=63 125	Employee ID Start/end date of absence

* `‘N`’ refers to the number of rows of the data set.

† See online supplemental file SI-1SI-1 for descriptions of individual JCTsjob cycle times.

‡ ’Conveyance’ refers to the transfer of a patient from the incident site to a hospital or equivalent location.

Two separate data sets (‘call handler’ and ‘paramedic’) were constructed from the historical assignment data set using the job type variable. Using the employee identification number as assigned in the Electronic Staff Record (ESR), the ‘call handlers’ and ‘paramedics’ historical assignment data sets were each aligned to the employment records, incidents, shift pattern, overtime and staff absence/sickness. For analysis, each data set by job type was subdivided into monthly units to create the time-series structure required for the time-varying covariates in accelerated failure time (AFT) and Cox proportional hazards (PH) analysis.

Prior to analysis, the demographic taxonomies were aggregated, combining values with low representation in the data set (see online supplemental file SI-2 for the transformations and frequencies). The operational variables of interest (staff absence, time spent on each aspect of job cycle time (JCT), Index of Multiple Deprivation (IMD) of incident location and acuity category of incidents) were each corrected for an exposure to allow for their relative size. ‘Time spent on each aspect of JCT’ and ‘incidents responded to by acuity category’ were corrected for the number of shifts worked in that month. ‘Time lost to absence’ was corrected for the relative length of the month (length of month in days, unless assignment began or was terminated during the month). The ‘jobs completed by IMD decile’ were converted to ‘percentage of incidents responded to within a given decile’. In the cases where no incidents were responded to in each month, for example, during onboarding for newly qualified paramedics, IMD percentages were imputed via mean imputation (first by employee ID and then the data set average should an employee have never responded to an incident).

Inclusion criteria were judged against unique ESR numbers based on employment history. Data were included for those who were employed and working as a call handler or paramedic between 1 January 2018 and 31 July 2023 (inclusive of limits), exclusive of individuals who had moved down in pay bands to commence the post. The data set comprised all variables as described above for the first two years of employment within the specific role (call handler or paramedic) within the study period (ie, an individual beginning a role on 1 July 2023 would have a censored observation after 31 July 2023). All data preparation was performed in R, making use of the ‘tidyverse’ framework.^15^ Data were analysed as these were recorded within the trust database systems and the period for data extraction was dictated by the data available at the trust.

### Data analysis

The analysis is interested in the effect of variables on time to event, and hence, data were analysed using time-varying covariates in AFT regression (a type of survival analysis) following testing and rejection of Cox PH regression (see online supplemental file SI-3 for Cox PH diagnostic tests). Usage of the Cox PH model where the proportional hazard assumption is not acceptable would have led to improper fitting of the model and incorrect inferences. AFT regression used the ‘aftreg’ function implemented in the ‘eha: Event History Analysis’ package.^16^ Six distributions were considered as parameterisations of the AFT model (‘Weibull’, ‘Gompertz’, ‘Extreme Value’, ‘Log-logistic’, ‘Log-normal’ and ‘Exponential’) with the optimal model selected using Bayesian information criteria (BIC) scores (see online supplemental file SI-4). For this analysis, an event is defined as an employee quitting their job, being fired or moved to a different role, and a non-event is when an employee remained in their role either as a call handler or paramedic.

As the intention of this study is to characterise what can be learnt from the available data, the sample size was not predetermined. To consider what sample size might be relevant to a properly powered study, a sample size of 796 individuals would be required to detect a 10% increase in odds for a step of 1 SD in a non-binary covariate (assuming a 10% attrition rate, 5% significance level and 80% power).^17^

### Patient and public involvement

Two lay representatives have been integral members of the research team, contributing to the project funding application, study design, delivery and dissemination. Two further lay representatives have been members of the project’s independent steering committee.

## Results

Table 2 provides a summary of the ambulance trust data by job type (call handler and paramedic), and percentages represent a proportion of the monthly data.

**Table 2:** Composition of the monthly data for call handler and paramedic analyses

Variable	Value	Monthly staff data	
		Call handlers	Paramedics
Staff demographics			
Age, years		31.0 (9.4)	28.0 (6.8)
Gender, %	Female	70.2%	52.2%
Nationality, %	British	89.7%	47.2%
	Not declared	2.1%	1.4%
	Other	8.2%	51.4%
Marital status, %	Divorced/legally separated/widowed	3.2%	1.9%
	Married/civil partnership	15.1%	13.2%
	Single	76.9%	80.3%
	Unknown	4.8%	4.6%
Staff pay scale			
Call handler, %	Band 3	64.8%	NA
	Band 4	35.2%	NA
Paramedic, %	Band 5	NA	96.0%
	Band 6+	NA	4.0%
Sickness/absence			
Staff absence duration (ratio of month)		0.069 (0.19)	0.039 (0.14)
Percentage of incidents attended in a month by IMD of incident location			
IMD: 1 (%)		NA	3.3 (4.2)
IMD: 2 (%)		NA	18.0 (10.0)
IMD: 3 (%)		NA	20.0 (10.0)
IMD: 4 (%)		NA	15.0 (7.7)
IMD: 5 (%)		NA	12.0 (7.1)
IMD: 6 (%)		NA	10.0 (6.8)
IMD: 7 (%)		NA	7.6 (6.1)
IMD: 8 (%)		NA	6.2 (5.5)
IMD: 9 (%)		NA	5.1 (5.9)
IMD: 10 (%)		NA	2.1 (3.8)
JCT, hours per shift worked			
‘Mobilisation’		NA	0.079 (0.058)
‘Running’		NA	0.62 (0.36)
‘On scene’		NA	3.1 (1.70)
‘To hospital‘		NA	0.62 (0.37)
‘Arrived at hospital to patient handover’		NA	0.82 (0.59)
‘Patient handover to clear’		NA	0.63 (0.40)
Non JCT time per shift worked		NA	3.20 (2.00)
Overtime (hours)			
Payment in lieu of break		NA	2.70 (3.10)
Planned		0.26 (2.3)	0.76 (2.20)
Unplanned		NA	0.20 (0.80)
Incident category per shift worked*			
Calls from people with life-threatening illnesses or injuries (Cat 1)		NA	0.37 (0.70)
Emergency calls (Cat 2)		NA	3.20 (1.90)
Urgent calls (Cat 3)		NA	1.20 (0.92)
Incidents (all categories) per shift worked		NA	5.10 (2.90)

Each term reports ‘mean (SD)’ unless otherwise stated.

* Calls of category 4 and above were removed from analysis due to rarity.

IMD, Index of Multiple Deprivation; JCT, job cycle time.

### Call handler staff

Data for 868 call handlers were analysed, comprising a total of approximately 925 years of combined employment. The average age for call handlers was 31 years (SD 9.4 years), 70.2% of the staff were recorded as female, 76.9% were single and 89.7% were declared as British nationals. In this study, 64.8% of call handlers were employed at ‘agenda for change’ band 3 and on average each call handler worked 15 minutes extra as planned overtime per month. Time lost due to absence and sickness averaged 6.9% of each month (approximately 2.1 days). Due to the nature of their work, call handler data did not include IMD, JCT and category of incidents per shift.

### Paramedic staff

Data for 1672 paramedics were analysed, comprising a total of approximately 2567 years of combined employment. The average age of the paramedic workforce was 28 years (SD 6.8 years), 52.2% of the staff were recorded as female, 80.3% were single and 47.2% declared as British nationals. In this study, 96.0% of paramedics were employed at band 5, and on average each worked 45.6 minutes extra as planned overtime and 12 minutes extra as unplanned overtime per month. Time lost due to absence and sickness among the paramedic staff averaged 3.9% of a month (approximately 1.2 days).

The paramedic staff responded to calls from a variety of locations representing different levels of deprivation as measured using the IMD; locations with IMD 2 and 3 recorded the highest percentage of incidents (18.0% and 20.0%, respectively), whereas locations with IMD 10 had the least percentage of incidents reported (2.1%).

During a shift, the paramedic spent most of their time actively responding to calls with this activity broken down into six ‘JCT’ descriptions (‘Mobilisation’, ‘Running’, ‘On scene’, ‘To hospital’, ‘Arrived at hospital to patient handover’ and ‘Patient handover to clear’, with full definitions given in online supplemental file SI-1). Among these, on average most of their time was spent ‘On scene’, that is, with/treating patients at the site of the incident, with the least time spent in ‘Mobilisation’. The time spent on shift not responding to an incident (eg, between incidents, attending meetings/training or performing maintenance) is ‘non-JCT time’, which accounted for 3.2 hours of each shift on average.

Each call that is made to the ambulance service is triaged and assigned a ‘categorisation’ according to a nationally devised description. The greater the risk to patient life, the higher the categorisation, with category 1 calls described as ‘Calls from people with life-threatening illnesses or injuries’. Which calls receive an ambulance response, and hence become ‘incidents’, is decided by the trust’s dispatch team with priority given based on the categorisation. On shift, the paramedics mostly experience category 2 incidents (‘Emergency calls’, 3.2 incidents per shift on average) and would be expected to respond to one category 1 incident (‘life-threatening illnesses or injuries’) for every three shifts worked (0.37 incidents per shift on average).

### Ambulance workforce turnover

Factors affecting the ambulance workforce turnover were identified using the AFT regression models considering time-varying covariates with the BIC metric selecting the ‘extreme value’ and ‘log-logistic’ families for the call handler and paramedic models, respectively. The results of both analyses are reported in tables3  4 for call handlers and paramedics, respectively.

**Table 3:** Summary of accelerated failure time* regression (‘extreme value’) for job type: call handler

Variable		Estimate	95% CI	P value†	Direction interpretation‡
Age		−0.0079	(−0.021, 0.006)	–	–
Gender	Male (reference)	–	–	–	–
	Female	0.29	(0.043, 0.54)	<0.05	… that time to leaving role increases if employee is female
Nationality	British (reference)	–	–	–	–
	Not declared	0.26	(−0.8, 1.30)	–	–
	Other	−0.38	(−0.74, −0.025)	<0.05	… that time to leaving role decreases if people do not identify as British
Marital status	Single (reference)	–	–	–	–
	Divorced/legally separated/widowed	0.28	(−0.51, 1.10)	–	–
	Married/civil partnership	0.00033	(−0.51, 1.1)	–	–
	Not declared	0.21	(−0.38, 0.80)	–	–
Pay scale	Band 3 (reference)	–	–	–	–
	Band 4	0.61	(0.33, 0.89)	<0.005	… that time to leaving role increases as pay increases
Staff absence duration (ratio of month)		−0.79	(−1.3, −0.3)	<0.05	… that time to leaving role decreases as time lost to short-term absences increases
Overtime: planned (hours)		0.16	(−0.065, 0.38)	–	–

* The AFTaccelerated failure time model is operating as a survival model in the implementation reported and hence a significant positive coefficient is indicative of an increased average survival time as the covariate increases.

† P value limits have been drawn from ‘An Introduction to Medical Statistics (Bland 2015)’.^29^

‡ Coefficients with p value>0.1 are represented by ‘-’.

**Table 4:** Summary of accelerated failure time* regression (‘log-logistic’) for job type: paramedic

Variable		Estimate	95% CI	P value†	Direction interpretation‡
Age		−0.0047	(−0.013, 0.0039)	–	–
Gender	Male (reference)				
	Female	0.061	(−0.038, 0.16)	–	–
Nationality	British (reference)				
	Not declared	−0.21	(−0.54, 0.13)	–	–
	Other	0.018	(−0.084, 0.12)	–	–
Marital status	Single (reference)				
	Divorced/legally separated/widowed	0.14	(−0.24, 0.52)	–	–
	Married/civil partnership	0.031	(−0.12, 0.18)	–	–
	Not declared	−0.16	(−0.38, 0.05)	–	–
Pay scale	Band 5 (reference)				
	Band 6+	−0.23	(−0.44, −0.019)	<0.05	… that time to leaving role decreases as pay increases
Staff absence duration (ratio of month)		0.04	(0.03, 0.06)	<0.005	… that time to leaving role increases as time lost to short-term absences increases
Overtime (hours)	Payment in lieu of breaks	0.28	(0.23, 0.34)	<0.005	… that time to leaving role increases for employees who work through breaks
	Planned	0.25	(0.18, 0.32)	<0.005	… that time to leaving role increases for employees who work planned overtime
	Unplanned	0.14	(−0.015, 0.3)	<0.1	… that time to leaving role increases where employees work unplanned overtime
Incidents (per shift worked)		0.18	(−0.13, 0.5)	–	–
Percentage of incidents attended in a month by IMD of incident location§	IMD: 1 (%)	0.0049	(−0.013, 0.023)	–	–
	IMD: 2 (%)	0.0042	(−0.0066, 0.015)	–	–
	IMD: 3 (%) (excluded)¶				
	IMD: 4 (%)	0.0083	(−0.0031, 0.02)	–	–
	IMD: 5 (%)	0.0006	(−0.01, 0.012)	–	–
	IMD: 6 (%)	−0.0037	(−0.015, 0.0072)	–	–
	IMD: 7 (%)	−0.0084	(−0.021, 0.004)	–	–
	IMD: 8 (%)	−0.028	(−0.038, −0.017)	< 0.005	… that time to leaving role decreases for employees who respond to more incidents at IMD:8 locales
	IMD: 9 (%)	0.014	(−0.00081, 0.03)	<0.1	… that time to leaving role increases for employees who respond to more incidents at IMD:9 locales
	IMD: 10 (%)	0.034	(0.0079, 0.06)	<0.05	… that time to leaving role increases for employees who respond to more incidents at IMD:10 locales
Job cycle time(hours per shift worked)	‘Mobilisation’	1.4	(−0.74, 3.6)	–	–
	‘Running’	0.29	(−0.4, 0.98)	–	–
	‘On scene’	0.18	(0.052, 0.31)	< 0.01	… that time to leaving role increases for employees who spend more time at the scene of incidents
	‘To hospital‘	−0.68	(−1.10, −0.22)	<0.005	… that time to leaving role decreases for employees who spend more time conveying patients
	‘Arrived at hospital to patient handover’	0.17	(−0.017, 0.36)	<0.1	…that time to leaving role increases for employees who spend more time waiting at hospitals
	‘Patient handover to clear’	0.2	(−0.18, 0.58)	–	–
	Non-JCT	0.097	(0.057, 0.14)	<0.005	… that time to leaving role increases for employees who spend more time outside JCT tasks
Incident category (per shift worked)	Calls from people with life-threatening illnesses or injuries (Category 1)	−0.33	(−0.64, −0.018)	<0.05	… that time to leaving role decreases for employees who respond to more ‘Category 1’ incidents
	Emergency calls (Category 2)	−0.32	(−0.63, −0.00)	<0.05	… that time to leaving role decreases for employees who respond to more ‘Category 2’ incidents
	Urgent calls (Category 3)	−0.068	(−0.42, 0.28)	–	–

* The AFTaccelerated failure time model is operating as a survival model in the implementation reported and hence a significant positive coefficient is indicative of an increased average survival time as the covariate increases.

† P value limits have been drawn from ‘An Introduction to Medical Statistics (Bland 2015)’.^29^

‡ Coefficients with p value>0.1 are represented by ‘-’.

§ IMDs are ordered from IMD:1 (highest levels of deprivation) to IMD: 10 (lowest levels of deprivations).

¶ IMD: 3 was removed from the analysis feature space to avoid over-specification of the model and was selected for removal as the most frequently attended IMD, and hence giving the most power as a reference category.

IMD, Index of Multiple Deprivation; JCT, job cycle time.

#### Factors impacting call handler turnover rates

Four factors were found to be associated with call handler workforce turnover: gender, nationality, pay scale and average absence duration. There was strong evidence to suggest that call handlers employed at band 4 were more likely to remain with their current employer longer (ie, a reduced risk of turnover) compared with those employed at band 3 (0.61, CI 0.33, 0.89, p value<0.005). A correlation between pay and retention was to be expected due to the pay progression structure of the ambulance trust; following their first-year employees move from band 3 to band 4 (with minimal exceptions), hence, increased retention time may not be due to the higher banding, but instead the higher banding is an outcome of retention. There is evidence to suggest female call handlers were less likely to leave compared with their male counterparts (0.29, CI 0.043, 0.54; p value<0.05). There was evidence to support a link between retention and call handler’s nationality and absence duration (p value<0.05); individuals who do not identify as ‘British’ have a higher risk of attrition, and individuals with an increased level of sick leave have a reduced probability of remaining in the service.

#### Factors impacting paramedic turnover rates

There was strong evidence to suggest that paramedic staff who worked more planned overtime or took sick leave were more likely to remain in service (p value<0.005). Likelihood to leave the service was, for the most part, unaffected by the level of deprivation (as measured using IMD deciles) associated with the location of the incident except for the lower IMDs (locations with low levels of deprivation). The data suggest attending to incidents in the least-deprived areas (IMD 9 and 10) reduces turnover, but attending to incidents in the next lowest (IMD 8) bracket increases turnover. Responding to high calls from people with life-threatening and emergency illnesses or injuries results in high paramedic turnover. With the increase of time spent on driving patients to hospital, there is a greater risk of attrition (−0.68, CI −1.10, −0.22; p value<0.005), whereas increased time spent at the scene of an incident, and between incidents (ie, non-JCT time), was linked to a reduced risk of attrition. There was evidence to suggest that paramedics employed at band 6 or above were more likely to leave compared with those employed at band 5 (−0.23, CI −0.44, −0.019, p value<0.05). However, none of the paramedic staff demographics (age, gender, marital status, nationality) were found to be associated with staff turnover.

## Discussion

The objective of this study was to identify the factors linked to staff retention/turnover among the call handler and paramedic workforce. While there are several studies involving emergency service workforce (including ambulance, fire and police services), there is a paucity of evidence into factors impacting staff retention/turnover among call handler and paramedic workforce within the UK. The complexities of retention, attrition and related concepts involve numerous factors influencing employee well-being and motivation.

The NHS, as the largest public employer, boasts a diverse demographic representation. This study found that the impact of demographic characteristics on workforce retention varied. Specifically, there was evidence linking gender and nationality to retention rates within the first two years of employment among call handlers. However, this trend was not observed in the paramedic workforce. The role of nationality aligns with findings reported by Moscelli *et al*,^18^ which highlighted that the impact of ethnicity on workforce retention was inconsistent across different clinical staff.^18^ The role of gender, notably that female staff remain in entry-level positions, reflects the concept of the ‘sticky floor’^19^ where women are less likely to move or pursue promotion or remain at the lower end of the pay scale perhaps due to fewer opportunities (if part-time) or responsibilities that limit their mobility such as childcare or caring for older adults. Therefore, any effective strategy to alleviate NHS workforce pressures, whether through retaining current employees or recruiting new ones, must be tailored to consider the diverse characteristics of the workforce, rather than adopting a one-size-fits-all approach.

This study has found an association between employees who take short-term sick or absence leave and a reduced risk of turnover in the paramedic workforce. An advantage of working for the NHS is that it provides paid sick leave for its employees, with the argument that paid sick leave reduces job instability associated with own or family member illness. The current study supports this argument; however, it only accounts for short-term sick leave. While extended sick leave might raise concerns about staff turnover, it is also plausible that a work environment that supports paid leave enables employees to attend to their own health needs or those of family members without risking their job security,^20 21^ hence are likely to stay longer with their current employer. More so, paid sick leave has previously been associated with job satisfaction in other professions such as nursing; job satisfaction linked with pay and benefits has also been found to correlate with intentions to remain within the EMS profession.^22 23^ There is evidence to suggest that burnout and stress are prevalent within the ambulance service environment associated with declining mental health, with some studies reporting more than 40% of the staff experiencing burnout.^10 24^ Burnout and stress may be the driving forces contributing to high sickness rates among the ambulance workforce compared with other professions within the UK NHS.^25^ A workplace culture that supports employees to attend to their own health needs can make a difference in the reduction of staff turnover, thus increasing workforce stability.

Constant demands, lengthy and extended shifts cause fatigue and exhaustion, symptoms of burnout, a condition commonly reported at a higher level in emergency services compared with other professionals in similar roles.^26^ Recent studies have shown burnout as a contributor for poor mental health which poses a threat to ambulance workforce retention. Burnout is a state of emotional, physical and mental exhaustion caused by prolonged stress linked to unsupportive management practices, long hours and physical demands of the paramedic role.^10^ While this study did not directly measure burnout and stress, time lost due to sickness, incident category and JCT have been considered as proxy measures. Therefore, the AFT models presented in this article mirror findings from other studies that suggest the link between ambulance staff burnout, stress and staff retention. For instance, paramedics attending higher numbers of the most stressful incidents (category 1 or 2) show an increase in their turnover risk which could suggest staff burnout. Interestingly, spending time between incident responses, reported as ‘non-JCT’ hours, shows a marked effect on retention (each hour per shift spent ‘not responding’ increases the average employment time by approximately 10% (95% CI 6%, 15%) within the first two years of employment). This suggests that factoring time between calls, allowing staff time to decompress or debrief before attending to their next job is beneficial for staff retention. In complex work environments, such as the ambulance sector, debriefing can serve as a valuable resource, enhancing team processes, promoting collaborative learning and contributing to staff well-being and resilience by mitigating burnout.^27^

An association between planned overtime variables and increased staff retention mirrors patterns from the wider staff satisfaction literature. Where overtime is voluntary and rewarded, as is the case for English paramedics, other studies have suggested a correlation with job satisfaction, and hence, retention.^28^ As the key mechanism here is that the overtime is voluntary, it would be improper to suggest that additional overtime would create retention. However, the uptake of voluntary overtime could be used as a proxy for satisfaction within the trust for proactive workforce planning. A reduction in voluntary overtime would be suggestive of reduced satisfaction, and the trust may want to either intervene to mediate the root cause or increase its recruitment.

Within the wider literature on retention, it is common to consider the role of each variable within Herzberg’s motivator-hygiene theory^12^ in order to discriminate between the themes of workplace ‘satisfaction’ and ‘dissatisfaction’. Within this context, we can examine how each feature of the model contributes to retention. Arguably, the ‘non-JCT’ time represents a mediator for hygiene risks in the workplace (eg, overwork) via both an implied limitation on work and by creating space for mental recovery. Hence, in settings where dissatisfaction is developing (eg, frozen salaries, policies lacking employee voice or poor administration), theoretically greater non-JCT time could mitigate the dissatisfaction. The inverse would also be possible, and a trust seeking to reduce non-JCT time while protecting staff retention levels should look to address workplace hygiene factors in tandem. We can consider the correlation between short-term sick leave and retention not as a factor, but as a proxy for, a positive work environment, and by contrast a lack of short-term sick leave may serve as a marker for subgroups of a trust that lack a supportive managerial environment.

The model presented for paramedics is more complex, and as a result more informative. This is not to say that call handlers could not benefit from an improved workplace, but rather that the data available for this study had greater limitations. Unfortunately, the workload of an individual call handler was not available in the current data reporting system operated at the trust, and so key variables around the acuity of their work, that is, the equivalent of incident categorisation and attendance, could not be included in the model.

This study has several strengths. It repurposes routinely collected operational data from an ambulance NHS Trust, including call handler and paramedic data, to investigate retention factors at an individual level. Compared with existing literature, which focuses more on qualitative methods, this study benefits from the ease of replicating the analysis or translating it to other trusts as it uses routinely collected data. The trust’s existing business intelligence and system administration teams often have the necessary skills to extract and interpret the data, while a statistician or data scientist can readily transform and structure it. Assuming the trust’s database systems remain static, such activities have an even lower barrier to replication at subsequent time points. This in-house activity has three key benefits: summarising chronic themes in the data, providing a mechanism to predict the ranking of attrition risks for individuals and allowing qualitative studies to focus on acute individualistic factors. However, this study was limited to a single ambulance trust, which may have resulted in missing pressures on older workforce members and those working in more rural or isolated environments. By developing the programme around routinely collected operational data sets, the process of transferring the analytical techniques, if not the findings, is relatively simple and could be used to inform workforce-centred retention strategies.

The analysis presented here has key limitations; the study focuses on a single ambulance trust which serves an area of high population density and is purely observational in nature. These facets mean the results may not generalise to other settings, either if the findings are applied at other trusts or if findings are acted on, the patterns detected may be either purely correlational or are the result of a causal latent variable which was absent from the model. However, due to the focus on readily available nationally agreed operational data which will have, if not an identical data structure, an equivalent in other trusts, the analytics can be readily mapped to new settings and used as data sources for follow-up on confirmational intervention studies. With respect to the data collection instruments, a strong limitation is the inclusion of ‘non-JCT’ time, which is a broad category with a significant contribution to the model. It is possible not all aspects of time spent outside the ‘JCT’ descriptions are of equal importance in driving retention, and further research in this area is vital.

## Conclusion

This study demonstrates that as pressures mount on the paramedic workforce it is key for workforce planners to allow for time between incidents for paramedics to reflect and recuperate should they wish to avoid high levels of attrition and burnout. The findings would suggest that while an overabundance of sick leave might be of traditional concern, an absence of sick leave among paramedic employees might serve as a warning that areas of the workplace lack a culture of trust and self-care which could lead to staff attrition. In addition, this study demonstrates a methodology for the extraction of novel knowledge from routinely collected operational data.

The transferability of the findings requires careful consideration. The underlying novel methodology to supplement our existing understanding of retention with data-driven predictions is generalisable to any healthcare setting which has readily available operational data. Considering the specific signals observed, features may generalise should the target environment have the core employee protections (eg, non-compulsory overtime with associated reward). Additionally, findings such as non-JCT and incident categorisation should be applied with nuance. Clearly a service needs to respond to the most intense incidents and operate as efficiently as possible, both to serve its population and create the satisfaction of a hard job well done. While several of the lessons found here could transfer well to other high-stress healthcare settings, with the accelerating pace of digital solutions in global healthcare a replication of the study to understand local drivers would often be more valuable. While the study findings highlight common drivers, they also point out specific predictors for retention among ambulance NHS workforce, thus underscoring the importance of workforce-centred retention strategies.

The focus of this study was within the initial two years of joining the trust due to the business priorities of the partner trust. Retaining new entries to the workforce is clearly key in high-pressure environments where the initial emotional shock of the job can lead to rapid burnout and attrition; however, maintaining staff past this point should not be overlooked. While this study has taken steps to explore these factors for the under-researched call handler group, the data available were strongly limited and future work is required to understand the impact of call acuity on staff satisfaction. In addition, this work has focused on analysis from the perspective of a new joiner to the trust, an equivalent analysis aiming to address likely attrition rates and the most likely group to protect/plan to replace given the current makeup of the workforce would be an invaluable tool for planning recruitment priorities.

## Supplementary material

10.1136/bmjopen-2024-098174 online supplemental file 1

## Data Availability

Data are available upon reasonable request.
