# Supplementary material for: AmReS: an observational retrospective time-to-event analysis of staff voluntary turnover in an English ambulance trust
Source: BMJ Open. 2025 Apr 15;15(4):e098174. doi: 10.1136/bmjopen-2024-098174 (PMC12001345; doi:10.1136/bmjopen-2024-098174)
Supplement: online supplemental file 1 [file bmjopen-15-4-s001.docx]

## SI-1 Paramedicine Terminology

Table SI-1A. ‘Job Cycle Time’ (JCT) descriptions.

| JCT term | Description |
| --- | --- |
| ‘Mobilisation’ | The time spent between being assigned a new incident and beginning to travel to the incident. |
| ‘Running’ | The time spent to travel to the site of an incident. |
| ‘On scene’ | The time spent at the incident site. |
| ‘To hospital‘ | [If patient conveyed]  The time spent transferring a patient from the incident to a hospital premises. |
| ‘Arrived at hospital to patient handover’ | [If patient conveyed]  The time spent waiting at hospital premises for the patient to be transferred to the hospitals care. |
| ‘Patient handover to clear’ | [If patient conveyed]  The time spent after the patient has been transferred to the hospitals care, e.g. completing medical notes and ensuring vehicle is prepared. |
| ‘Non-JCT Activity’ | Activities undertaken on shift that are outside the bounds of the ‘JCT’. Examples include time spent:   - between dispatched incidents - on maintenance - in meetings - in training |

## SI-2 Demographic taxonomy tables

Summary of demographic taxonomies as found in the supplied data (‘Original’) as opposed to the aggregated terms used in analysis (‘Transformed’), with frequencies based on head count.

Table SI-2A: Paramedic Gender distribution

| Original | Frequency | Percentage |
| --- | --- | --- |
| Male | 798 | 47.7 |
| Female | 874 | 52.3 |
| Total | 1672 | 100.0 |

Table SI-2B: Paramedic Nationality distribution

| Original | Frequency | Percentage | Transformed | Frequency | Percentage |
| --- | --- | --- | --- | --- | --- |
| British | 795 | 47.5 | British | 795 | 47.5 |
| Australian | 641 | 38.3 | Others | 853 | 51.0 |
| Irish | 40 | 2.4 |  |  |  |
| Namibian | 28 | 1.7 |  |  |  |
| New Zealander | 31 | 1.9 |  |  |  |
| Nigerian | 10 | 0.6 |  |  |  |
| South African | 39 | 2.3 |  |  |  |
| Others* | 64 | 3.8 |  |  |  |
| Not Declared | 24 | 1.4 | Not Declared | 24 | 1.4 |
| Total | 1672 | 100.0 | Total | 1672 | 100.0 |

Table SI-2C: Paramedic Distribution of marital status

| Original | Frequency | Percentage | | | Transformed | Frequency | Percentage |
| --- | --- | --- | --- | --- | --- | --- | --- |
| Single | 1334 | | 79.8 | Single | | 1334 | 79.8 |
| Divorced | 28 | | 1.7 | Divorced/Legally Separated/ Widowed | | 35 | 2.1 |
| Legally Separated | 6 | | 0.4 |  |  |  |  |
| Widowed | 1 | | 0.1 |  |  |  |  |
| Married | 171 | | 10.2 | Married/Civil Partnership | | 219 | 13.1 |
| Civil Partnership | 48 | | 2.9 |  |  |  |  |
| Unknown | 74 | | 4.4 | Not Declared | | 84 | 5.0 |
| Missing | 10 | | 0.6 |  |  |  |  |
| Total | 1672 | | 100.0 | Total | | 1672 | 100.0 |

Table SI-2D: Paramedic Pay scale distribution

| Original | Frequency | Percentage | Transformed | Frequency | Percentage |
| --- | --- | --- | --- | --- | --- |
| XR05 | 1510 | 90.3 | Band 5 | 1510 | 90.3 |
| XR06 | 157 | 9.4 | Band 6+ | 162 | 9.7 |
| XR07 | 5 | 0.3 |  |  |  |
| Total | 1672 | 100.0 | Total | 1672 | 100.0 |

Table SI-2E: Call handler Gender distribution

| Gender | Frequency | Percentage |
| --- | --- | --- |
| Male | 267 | 30.7 |
| Female | 601 | 69.3 |
| Total | 868 | 100.0 |

Table SI-2F: Call handler Nationality distribution

| Original | Frequency | Percentage | Transformed | Frequency | Percentage |
| --- | --- | --- | --- | --- | --- |
| British | 763 | 87.9 | British | 763 | 87.9 |
| Australian | 11 | 1.3 | Others | 88 | 10.1 |
| Irish | 15 | 1.7 |  |  |  |
| Nigerian | 10 | 1.2 |  |  |  |
| Others* | 52 | 6.0 |  |  |  |
| Not Declared | 17 | 2.0 | Not Declared | 17 | 2.0 |
| Total | 868 | 100.0 | Total | 868 | 100.0 |

Table SI-2G: Call handler Distribution of marital status

| Original | Frequency | Percentage | Transformed | Frequency | Percentage |
| --- | --- | --- | --- | --- | --- |
| Single | 660 | 76.0 | Single | 660 | 76.0 |
| Divorced | 19 | 2.2 | Divorced/Legally Separated/ Widowed | 23 | 2.6 |
| Legally Separated | 3 | 0.3 |  |  |  |
| Widowed | 1 | 0.1 |  |  |  |
| Married | 112 | 12.9 | Married/Civil Partnership | 130 | 15.0 |
| Civil Partnership | 18 | 2.1 |  |  |  |
| Unknown | 32 | 3.7 | Not Declared | 55 | 6.3 |
| Missing | 23 | 2.6 |  |  |  |
| Total | 868 | 100.0 | Total | 868 | 100.0 |

Table SI-2H: Call handler Pay scale distribution

| Original | Frequency | Percentage | Transformed | Frequency | Percentage |
| --- | --- | --- | --- | --- | --- |
| XN03 | 440 | 50.7 | Band 3 | 440 | 50.7 |
| XN04 | 428 | 49.3 | Band 4 | 428 | 49.3 |
| Total | 869 | 100.0 | Total | 869 | 100.0 |

## SI-3 Cox Proportional Hazard Assumption Testing

Performed using the ‘cox.zph’ function implemented in ‘survival’ which follows the diagnostics recommended by Grambsch and Therneau.

Table SI-3A. Cox PH diagnostic summaries for Paramedic data. A low p-value is evidence to reject the assumption of proportionality.

| **Variable** | | **ChiSq** | **Df** | **p-value Interpretation** |
| --- | --- | --- | --- | --- |
| Age |  | 0.70 | 1 | - |
| Gender |  | 0.01 | 1 | - |
| Nationality |  | 8.84 | 2 | Moderate evidence (< 0.05) |
| Marital Status |  | 14.41 | 3 | Strong evidence (< 0.005) |
| Pay Scale |  | 4.21 | 1 | Moderate evidence (< 0.05) |
| Staff Absence Duration |  | 1.29 | 1 | - |
| Over time (hours) | Payment in lieu of break | 17.28 | 1 | Strong evidence (< 0.005) |
|  | Planned | 7.10 | 1 | Good evidence (< 0.01) |
|  | Unplanned | 7.85 | 1 | Good evidence (< 0.01) |
| Incidents (per Shift Worked) |  | 7.12 | 1 | Good evidence (< 0.01) |
| Incident by response location IMD (% of incidents attended) | IMD: 1 | 1.04 | 1 | - |
|  | IMD: 2 | 0.04 | 1 | - |
|  | IMD: 3 (excluded) |  |  |  |
|  | IMD: 4 | 0.15 | 1 | - |
|  | IMD: 5 | 2.10 | 1 | - |
|  | IMD: 6 | 6.64 | 1 | Good evidence (< 0.01) |
|  | IMD: 7 | 0.09 | 1 | - |
|  | IMD: 8 | 0.36 | 1 | - |
|  | IMD: 9 | 1.11 | 1 | - |
|  | IMD: 10 | 0.00 | 1 | - |
| Job Cycle Time  (Hours per Shift Worked) | “mobilisation” | 5.86 | 1 | Moderate evidence (< 0.05) |
|  | “running” | 5.15 | 1 | Moderate evidence (< 0.05) |
|  | “on scene” | 6.57 | 1 | Moderate evidence (< 0.05) |
|  | “to hospital” | 3.18 | 1 | Weak evidence (< 0.1) |
|  | “arrived at hospital to patient handover” | 5.04 | 1 | Moderate evidence (< 0.05) |
|  | “patient hand over to clear” | 5.25 | 1 | Moderate evidence (< 0.05) |
|  | Non JCT | 9.19 | 1 | Strong evidence (< 0.005) |
| Incident Category (per shift worked) | Calls from people with life-threatening illnesses or injuries (Cat 1) | 0.28 | 1 | - |
|  | Emergency calls (Cat 2) | 6.11 | 1 | Moderate evidence (< 0.05) |
|  | Urgent calls (Cat 3) | 7.39 | 1 | Good evidence (< 0.01) |

Table SI-3B. Cox PH diagnostic summaries for Call handler data. A low p-value is evidence to reject the assumption of proportionality*.

| **Variable** | | **ChiSq** | **Df** | **p-value Interpretation** |
| --- | --- | --- | --- | --- |
| Age |  | 14.36 | 1 | Strong evidence (< 0.005) |
| Gender |  | 0.02 | 1 | - |
| Nationality |  | 0.48 | 2 | - |
| Marital Status |  | 2.06 | 3 | - |
| Pay Scale |  | 0.04 | 1 | - |
| Staff Absence Duration |  | 8.41 | 1 | Strong evidence (< 0.005) |
| Over time (hours) | Planned | 1.47 | 1 | - |

*P. Grambsch and T. Therneau (1994), Proportional hazards tests and diagnostics based on weighted residuals. Biometrika, **81**, 515-26.

## SI-4 AFT Family decision metrics

Table SI-4. Summary of AIC and BIC values for Paramedic and Call handler AFT models with each distribution family. The families in bold are the optimal BIC, and hence the family selected for reporting.

| Job Type | Family | AIC | BIC |
| --- | --- | --- | --- |
| Call handler | Weibull | 5516 | 5606 |
|  | Gompertz | 5541 | 5631 |
|  | **Extreme value** | **5513** | **5604** |
|  | Log-logistic | 5517 | 5607 |
|  | Log-normal | 5530 | 5621 |
|  | Exponential | 5539 | 5621 |
| Paramedic | Weibull | 4183 | 4472 |
|  | Gompertz | 4402 | 4691 |
|  | Extreme value | 4238 | 4527 |
|  | **Log-logistic** | **4110** | **4399** |
|  | Log-normal | 4380 | 4669 |
|  | Exponential | 4425 | 4705 |
