# Supplementary material for: AmReS: an observational retrospective time-to-event analysis of staff voluntary turnover in an English ambulance trust
Source: BMJ Open. 2025 Apr 15;15(4):e098174. doi: 10.1136/bmjopen-2024-098174 (PMC12001345; doi:10.1136/bmjopen-2024-098174)
Supplement: Supplementary file 2 [file bmjopen-2024-098174.reviewer_comments.pdf]

PEER REVIEW HISTORY

BMJ Open publishes all reviews undertaken for accepted manuscripts. Reviewers are asked to complete a checklist review form and are provided with free text boxes to elaborate on their assessment. These free text comments are reproduced below.

ARTICLE DETAILS

Title (Provisional)

AmReS - an observational retrospective time- to-event analysis of staff voluntary turnover in an English ambulance trust

Authors

Cook, Robert; Shabuz, Zillur Rahman; Bennett, Matthew; East, Abigail; Dube, Alisen; Miller, Josh; Asaduzzman, Md; Radford, Mark; Leary, Alison; Jones, Sarahjane

---

VERSION 1 - REVIEW

**Reviewer**	**1**
**Name**	**Weiss, Katja**
**Affiliation**	**University of Zurich, Institute of Primary Care**
**Date**	**01-Jan-2025**
**COI**	**None**

General

Use the same date formatting. Some cases are YYYY/YY and some YYYY/YYYY (eg. P3 L51-L56)

Introduction

P3 L47-48: Only the number of calls doesn't provide any information. You have to include the number of emergency call handlers to convey relevance. Also, there is no reference for your statement. Reference #10 only provides information for the Yorkshire Ambulance Service between July 2019 and September 2019.

P4 L10-L20: This paragraph should be reworked because it features multiple unrelated statements. Also the sentence: "In December 2022, the NHS Ambulance service for England reported a 60%

increase in the number of calls received over a one-week period compared to the previous

week and the highest number on record excluding the beginning of the COVID-19 pandemic

[5]." Why is comparing one week and the previous week in December relevant? I would assume that the week with more calls has to do something with the holidays, which should be a yearly occurrence and not a new observation.

Methods

P7 L37: Truts database system -> Trust database system

Discussion

The discussion primarily focuses on the paramedics and only mentions call handlers in one paragraph with very little insight. Did the call handlers also experience burnout? How many calls does one call handler take on average?

Conclusion

Can any conclusions be extracted from the call handler analysis? Are there any ways to address the attrition rates for the call handlers?

**Reviewer**	**2**
**Name**	**Aungsuroch, Yupin**
**Affiliation**	**Chulalongkorn University**
**Date**	**11-Jan-2025**
**COI**	**None**

Abstract:

Highlight the most significant results only.

Align interpretative conclusions with data-driven insights.

Summarize or omit the "Strengths and Limitations" section.

Introduction:

Avoid repetitive content and excessive statistics.

Expand the discussion on call handlers for narrative balance.

Clearly identify gaps in previous retention research and how this study addresses them.

Methods:

Move granular details (e.g., imputation methods) to supplementary materials.

Explain the statistical rationale for AFT regression and its implications.

Clarify exclusion criteria to mitigate concerns about selection bias.

Detail the role of patient/public involvement.

Results:

Add context to significant findings, like the psychological impact of Category 1 and 2 calls or organizational culture's role in retention.

Explore variability in data (e.g., standard deviations, skewness) for nuanced trends.

Support patterns (e.g., gender, nationality differences) with qualitative data or literature.

Discussion:

Provide detailed insights into key findings, such as turnover linked to IMD or overtime.

Address systemic factors like NHS policies or organizational culture affecting retention.

Relate findings to workforce retention theories (e.g., Herzberg's motivation-hygiene theory).

Conclusion:

Offer specific workforce management recommendations (e.g., mental health support, flexible working hours).

State study limitations and propose areas for future research.

Discuss the findings' generalizability to other healthcare settings or countries.

VERSION 1 - AUTHOR RESPONSE

Reviewer 1

Use the same date formatting. Some cases are YYYY/YY and some YYYY/YYYY (eg. P3 L51-L56)

Thank you for spotting this discrepancy. It has been fixed and we believe the manuscript is now consistent.

P3 L47-48: Only the number of calls doesn't provide any information. You have to include the number of emergency call handlers to convey relevance. Also, there is no reference for your statement. Reference #10 only provides information for the Yorkshire Ambulance Service between July 2019 and September 2019.

P4 L10-L20: This paragraph should be reworked because it features multiple unrelated statements. Also the sentence: "In December 2022, the NHS Ambulance service for England reported a 60% increase in the number of calls received over a one-week period compared to the previous week and the highest number on record excluding the beginning of the COVID-19 pandemic

" Why is comparing one week and the previous week in December relevant? I would assume that the week with more calls has to do something with the holidays, which should be a yearly occurrence and not a new observation.

The introduction has been heavily reworked in combination with reviewer 2s comments. We believe all of these sections have been removed or reworked for better flow and welcome any new comments.

Methods: P7 L37: Truts database system -> Trust database system

Fixed.

Discussion: The discussion primarily focuses on the paramedics and only mentions call handlers in one paragraph with very little insight. Did the call handlers also experience burnout? How many calls does one call handler take on average?

The data taken from the Trust was unfortunately lacking a link between contacts and the call handler who dealt with them, and hence there are few signals to discuss. The present lack of a link was a frustrating limitation of the data. We believe given the limited attention call handlers receive, reporting the current study of call handlers alongside the paramedic analysis is preferable to omission. We have added a paragraph to the discussion to highlight this issue.

Commented [SJ1]: I've had to read this sentence a couple of times. Rephrase for ease of reading.

Conclusion: Can any conclusions be extracted from the call handler analysis? Are there any ways to address the attrition rates for the call handlers?

Unfortunately, the signals that have arisen for the call handlers are outside of a Trusts control. E.g. we would not suggest they hire only female call handlers to improve retention. We have expanded the recommendations to suggest a more in depth analysis of call handler activity is needed.

Reviewer 2

Abstract:

Highlight the most significant results only.

We are unsure of the exact meaning of most significant here - we have chosen to report those with the highest operational significance of those with a statistically significant result.

Commented [SJ2]: Didn't we make some changes to this? Perhaps be clear about that to say we address it?

Commented [RC3R2]: We haven't changed the signals we report - but have added the coefficient and CIs.

Align interpretative conclusions with data-driven insights.

We have updated the conclusion section to more explicitly link result to conclusion.

Summarize or omit the "Strengths and Limitations" section.

We are following the BMJ open structure which asks for this section as is, and politely decline to follow this comment.

Introduction:

The introduction has been extensively reworked given your insightful comments. We believe this captures many of the comments you made and are open to any feedback.

Methods:

Move granular details (e.g., imputation methods) to supplementary materials.

Following the editors advice we have politely declined to do this.

Explain the statistical rationale for AFT regression and its implications.

Brief statements have been added to the methods section to explain why Cox PH was rejected and hence AFT selected/

Clarify exclusion criteria to mitigate concerns about selection bias.

The inclusion criteria is there to specify a population, of whom we selected all employees. As a result we don't believe there is selection bias as we work with this population but welcome clarity as to the reviewers meaning.

Detail the role of patient/public involvement.

The section on PPIEP already does this, can the reviewer please be clearer as to what they require.

Results:

Add context to significant findings, like the psychological impact of Category 1 and 2 calls or organizational culture's role in retention.

This is done in the discussion where we believe it is more appropriate

Explore variability in data (e.g., standard deviations, skewness) for nuanced trends.

This is not the intention of the article, or critical to answering the research question. It would also run the risk of introducing multiplicity should we update the variable space at this late stage and hence we have declined to do it.

Support patterns (e.g., gender, nationality differences) with qualitative data or literature.

Reflection on how the signals take shape in the wider literature is done in the discussion section where we believe it is more appropriate.

Discussion:

Provide detailed insights into key findings, such as turnover linked to IMD or overtime.

We have added a paragraph reflecting on overtime, however discussion of IMD feels highly hypothetical.

Address systemic factors like NHS policies or organizational culture affecting retention.

As a single site study the policies and organisational factors are static, we have no evidence as to how varying them would effect retention and so we feel it is outside the remit of this paper

Relate findings to workforce retention theories (e.g., Herzberg's motivation-hygiene theory).

We have expanded the discussion to draw on Herzberg's theory and put the results into context. This study is more aiming to characterize the behaviours as seen and we feel to go deeper into theoretical possibilities would be hypothetical and should be left to systematic review and meta analysis.

Conclusion:

Offer specific workforce management recommendations (e.g., mental health support, flexible working hours).

We have not studied any particular mechanism so this work does not give evidence for what does and doesn't work, hence this would be highly hypothetical and as such we politely decline to add this.

State study limitations and propose areas for future research.

We have left the limitations in the discussion as we believe they fit better here. We have also expanded the section of the conclusion that recommend further work to better highlight the need for more nuanced analysis of the call handler group.

Discuss the findings' generalizability to other healthcare settings or countries.

We have expanded the discussion of generalizability but do not want to draw strong conclusions outside the space of the study.
